# Development of a Numerical Model to Predict the Dielectric Properties of Heterogeneous Asphalt Concrete

**DOI:** 10.3390/s21082643

**Published:** 2021-04-09

**Authors:** Qingqing Cao, Imad L. Al-Qadi

**Affiliations:** Illinois Center for Transportation, University of Illinois at Urbana-Champaign, Rantoul, IL 61866, USA; qc13@illinois.edu

**Keywords:** ground-penetrating radar, asphalt pavement, finite-difference time-domain modeling, heterogeneous model

## Abstract

Ground-penetrating radar (GPR) has been used for asphalt concrete (AC) pavement density prediction for the past two decades. Recently, it has been considered as a method for pavement quality control and quality assurance. A numerical method to estimate asphalt pavement specific gravity from its dielectric properties was developed and validated. A three-phase numerical model considering aggregate, binder, and air void components was developed using an AC mixture generation algorithm. A take-and-add algorithm was used to generate the uneven air-void distribution in the three-phase model. The proposed three-phase model is capable of correlating pavement density and bulk and component dielectric properties. The model was validated using field data. Two methods were used to calculate the dielectric constant of the AC mixture, including reflection amplitude and two-way travel time methods. These were simulated and compared when vertical and longitudinal heterogeneity existed within the AC pavement layers. Results indicate that the reflection amplitude method is more sensitive to surface thin layers than the two-way travel time methods. Effect of air-void content, asphalt content, aggregate gradation, and aggregate dielectric constants on the GPR measurements were studied using the numerical model.

## 1. Introduction

Ground-penetrating radar (GPR) is a nondestructive testing method widely applied for monitoring and assessing civil structures. In pavement engineering, GPR data are used to predict pavement density and layer thickness, and to detect anomalies underneath pavement surfaces [1,2,3].

For asphalt concrete (AC), pavement, density is a key factor that affects pavement performance and service life. It is an important indicator of AC quality control and quality assurance during compaction. Predicting AC density can avoid under or over compaction, and it ensures the designed density level is achieved. Among available nondestructive techniques, GPR is deemed to be cost- and time-effective, and could be used to predict AC density or air void content during compaction [4,5,6,7]. Compared with other destructive or nondestructive test methods, GPR has the advantage of performing potential high-accuracy measurements, a large-coverage area, and relatively high-speed surveys.

Numerical modeling and simulation of GPR responses can help in better understanding electromagnetic (EM) waves interaction with AC pavements [8]. Compared with constructing physical AC pavement test mats and performing tests on them, numerical simulation is time- and cost-efficient. Apart from that, the structure and material properties of the AC mixture are better controlled with numerical modeling. Finite-difference time-domain (FDTD) method, a powerful tool for numerical modeling, is an appropriate tool for EM wave propagation simulation [9]. Although most of the research using the FDTD method has focused on the detection of underground objects, such as faults and caves, tunnel inspections, pipes, and landmines [10,11,12,13], this method has recently been applied to bridge assessment and inspection as well as pavement surface-moisture removal [14,15,16]. Microstructure and density of AC pavement research using the FDTD method, however, is still limited.

Civil engineering materials, such as AC mixture, concrete, stone, and bricks, have been considered homogeneous materials in the GPR numerical modeling application [8,17,18,19,20]. This, however, is a simplification of material dielectric properties. On the other hand, the heterogeneity of a material causes an impact on GPR measurements at two levels. First, the material properties show variability along space. This arises from the internal configuration and distribution of particles that compose the material [21]. Second, the received signals from the material are uncertain as they come from the randomness of the generated heterogeneous models. Most FDTD simulations of heterogenous material studies have concentrated on soil, which is achieved by defining a series of dispersive material properties in the FDTD model [22].

Apart from soil, Benedetto et al. used a random-sequential absorption algorithm to generate random 2D distributions of the compacted ballast aggregates to assess railway ballast condition [23]. Lachowicz and Rucka proposed a heterogeneous numerical model to simulation concrete, and the model was validated through laboratory tests [24]. No specific method, however, has been proposed for modeling AC mixture as a heterogeneous material using the FDTD method.

The AC mixture can be viewed as a three-phase material, including aggregate, asphalt binder, and air voids. Although not yet reported in the FDTD methods, the numerical model of the AC mixture has been widely used in research using the discrete-element and finite-element methods [25,26,27]. Computed tomography (CT) scan reconstruction and random aggregate generation are two commonly used methods to generate the heterogeneous microstructure of AC [27]. The CT scan method uses real specimen images either prepared in the laboratory or cut from constructed pavement. Many specimens are usually needed to capture the variation of microstructures. The random aggregate generating method generates the microstructure of AC by randomly distributing aggregates with different sizes. It is more suitable for batch modeling and parameter control.

In this paper, a three-phase numerical model was developed considering aggregate, binder, and air void components. The simulation results were compared with results from theoretical pavement density prediction models, and the model was validated using field tests. A take-and-add algorithm was further proposed to randomly generate the non-uniform air-void distribution in the three-phase model. Sensitivity analyses were performed, and the effect of air-void and asphalt binder contents as well as aggregate gradation and their corresponding dielectric constants predicted from GPR measurements were studied. Two methods, reflection amplitude and two-way travel time methods, were used to calculate the AC dielectric constant from simulation results. The results were then compared for cases of vertical and longitudinal heterogeneity within an AC pavement layer.

## 2. Background on AC Pavement Density Prediction

### 2.1. Pavement Dielectric Constant Calculation

For AC pavement density prediction from GPR data, sent and reflected signals from the interface of two materials with different dielectric properties are used in the analysis. The signals can be used to compute pavement dielectric constants, which can then be related to AC densities through theoretical or empirical formulas. The reflection amplitude and two-way travel time (TWTT) methods were usually used to calculate the dielectric constants of pavement. The reflection amplitude method utilizes Fresnel equations, and it is widely used for the analysis of field test results because it does not need ground truth (GT) cores [16]. The TWTT method, on the other hand, allows the calculations of the material’s dielectric constant from the average velocity of EM waves in the material. It is usually used to back-calculate a pavement layer’s thickness [28].

#### 2.1.1. Reflection Amplitude Method

**Figure 1:**
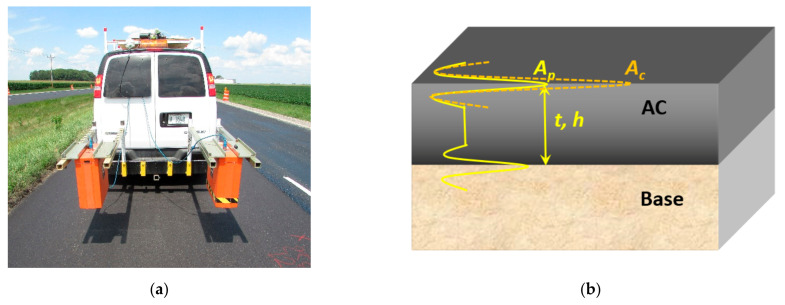
The ground-penetrating radar (GPR) survey configuration and received signal of GPR from field tests (**a**) and reflected signal from interface of layers (**b**).

In the reflection amplitude method, air-coupled antennas are installed at a specific distance from the pavement surface (see Figure 1a). EM waves are sent into the ground by a transmitter antenna, and the reflected waves are received by a receiver antenna. When the EM wave propagates through the AC pavement, part of its energy is reflected at interfaces, such as between the air and pavement surface layer (the top of the AC layer) and between the AC layer and the base layer (see Figure 1). To calculate a pavement’s dielectric constants using the reflection amplitude method, two GPR surveys need to be performed. One survey is on the pavement surface, where the amplitude of the EM wave reflected from the top of the AC layer is recorded as $A_{0}$. Another survey is performed on a complete perfect electricity conductor, usually a metal (e.g., copper) plate which covers the antenna’s footprint. The amplitude of the pulse reflected from the metal plate is recorded as $A_{c}$. The relative dielectric constant, ϵ, of the pavement surface can be calculated using Equation (1).

(1) $$\epsilon =\;{\left(\frac{1+\frac{A_{p}}{A_{c}}}{1-\frac{A_{p}}{A_{c}}}\right)}^{2}$$

#### 2.1.2. TWTT Method

In the TWTT method, the dielectric constant is calculated within the material. The travel speed of the EM wave in a material is decided by the material’s dielectric property. The speed of the EM wave can be calculated using the known pavement layer thickness, h, and the measured time interval, t,  of pulses reflected from the top, $A_{0},$ and bottom of the pavement layer, $A_{1}$. The formula of the TWTT method is shown in Equation (2). Because the TWTT method uses the average speed of the EM wave within the material to be measured, the dielectric constant measured using this method is the average, or bulk dielectric constant, of the material

(2) $$\epsilon ={\left(\frac{c*h}{2t}\right)}^{2}$$

Here c is the speed of light.

### 2.2. Theoretic EM Mixing Models and Pavement Density Prediction

Once the pavement’s dielectric constant is obtained, it can be related to the AC’s densities through theoretical equations. Three mixing models, the Al-Qadi-Lahouar-Leng (ALL) model, complex refractive index model (CRIM), and Bottcher model (see Equations (3)–(5)), are derived from the EM mixing theory [7,29]. The EM mixing theory relates the dielectric constant of a mixture, $\epsilon_{AC},$ to the dielectric and volumetric properties of its components. For dry AC pavement, three phases—aggregate, asphalt binder, and air—are considered in these models.

(3) $$\frac{\epsilon_{\mathrm{AC}}-\epsilon_{b}}{3\epsilon_{AC}-2.3\epsilon_{b}}=V_{se}\frac{\epsilon_{s}-\epsilon_{b}}{\epsilon_{s}+2\epsilon_{AC}-2.3\epsilon_{b}}+V_{a}\frac{\epsilon_{a}-\epsilon_{b}}{\epsilon_{a}+2\epsilon_{AC}-2.3\epsilon_{b}}\;$$

(4) $$\frac{\epsilon_{\mathrm{AC}}-\epsilon_{b}}{3\epsilon_{AC}}=V_{se}\frac{\epsilon_{s}-\epsilon_{b}}{\epsilon_{s}+2\epsilon_{AC}}+V_{a}\frac{\epsilon_{a}-\epsilon_{b}}{\epsilon_{a}+2\epsilon_{AC}}$$

(5) $$\sqrt{\epsilon_{AC}}=V_{a}\sqrt{\epsilon_{a}}+V_{se}\sqrt{\epsilon_{s}}+V_{b}\sqrt{\epsilon_{b}}$$

Here $V_{se}$ is effective volume of aggregate, and $V_{a}$ is volume of air. $\epsilon_{b}$ is the dielectric constant of asphalt binder, which is usually set as 3. $\epsilon_{a}$ is the dielectric constant of air, which is usually set as 1. $\epsilon_{s\;}$ is the aggregate dielectric constant, which can be back-calculated from field cores or obtained from a database.

Substituting equations from the volumetric properties of the AC mixture in Equations (3)–(5) yields the ALL and modified CRIM and Bottcher specific gravity models (see Equations (6)–(8)). The bulk specific gravity, $G_{mb}$, of the AC mixtures value can be determined.

(6) $$G_{mb}=\frac{\frac{\varepsilon_{AC}-\varepsilon_{b}}{3\varepsilon_{AC}-2.3\varepsilon_{b}}-\frac{1-\varepsilon_{b}}{1-2.3\varepsilon_{b}+2\varepsilon_{AC}}}{\frac{\varepsilon_{s}-\varepsilon_{b}}{\varepsilon_{s}-2.3\varepsilon_{b}+2\varepsilon_{AC}}\;\frac{1-P_{b}}{G_{se}}-\frac{1-\varepsilon_{b}}{1-2.3\varepsilon_{b}+2\varepsilon_{AC}}\;\frac{1}{G_{mm}}}$$

(7) $$G_{mb}=\frac{\sqrt{\varepsilon_{AC}}-1}{\;\frac{P_{b}}{G_{b}}\sqrt{\varepsilon_{b}}+\frac{1-P_{b}}{G_{se}}\sqrt{\varepsilon_{s}}-\frac{1}{G_{mm}}}$$

(8) $$G_{mb}=\frac{\frac{\varepsilon_{AC}-\varepsilon_{b}}{3\varepsilon_{AC}}-\frac{1-\varepsilon_{b}}{1+2\varepsilon_{AC}}}{\frac{\varepsilon_{s}-\varepsilon_{b}}{\varepsilon_{s}+2\varepsilon_{AC}}\;\frac{1-P_{b}}{G_{se}}-\frac{1-\varepsilon_{b}}{1+2\varepsilon_{AC}}\;\frac{1}{G_{mm}}}$$

Here $G_{mm}$ is the maximum specific gravity of the AC mixture, and $G_{se}$ is the effective specific gravity of the aggregates. $P_{b}$ is the binder content of the AC mixture. These AC volumetric values can be obtained from the plant prior to pavement compaction. With the dielectric constant of a mixture, $\epsilon_{AC}$, calculated from Equations (1) or (2), the bulk specific gravity of the AC mixture, $G_{mb}$, can be determined for in-situ AC.

A one-phase pavement model developed by Shangguan and Al-Qadi [8] was used to simulate AC. The dielectric constants of the AC mixture and base layers are preset; each as a single value. The one-phase model was successful in simulating EM waves inside dry AC pavement and studying the effect of surface moisture on the GPR signal. However, the model simplifies the AC mixture as a homogeneous material, limiting the relationship between the AC mixture’s dielectric constant and the volumetric properties of its components. In the proposed three-phase model, the dielectric constant and volumetric properties of air, asphalt binder, and aggregates in the AC are considered.

## 3. Methodology

### 3.1. Finite-Difference Time-Domain Simulation

The finite-difference time-domain (FDTD) simulation in this study was performed using an open-source GPR simulation program known as GprMax [30]. It has been successfully applied to simulate GPR wave propagation in various materials. The FDTD method, also known as Yee’s algorithm, is a differential equation-based solver that provides numerical solutions for Maxwell’s equations in complex geometries [31]. The FDTD method uses the second order accurate derivatives in space and time. It utilizes a mesh built from rectangular, or Yee cells, in which field values are updated time-step by time-step as EM waves propagate through a structure. In this paper, 2D FDTD simulations were performed considering the computational intensity of 3D simulations. Two-dimensional FDTD simulation has been proven to have similar results to 3D simulations in the case of AC pavement [8].

### 3.2. Generating Three-Phase Structure of Asphalt Mixture Models

In this paper, AC mixture was assumed dry. Asphalt binder, aggregate, and air voids compose the three-phase heterogeneous AC mixture. Compared with the Computed tomography (CT) scan method, the random aggregate generating method does not depend on the microstructure of any existing specimens. Thus, it is more suitable for batch modeling and parameter control. In this study, coarse aggregates were generated as circles of different sizes. Considering the cubic meshes in the FDTD method, these circles were later approximated using unit squares of 0.001 × 0.001 m to acquire the required level of accuracy. The angularity of particles was not considered in this study because the angularity of aggregates mainly affects the mechanical properties of the material rather than its dielectric properties. The three-phase AC mixture generation algorithm is summarized as follows:

1. Choose a mean size between adjoining sieve sizes as aggregate size in each level.
2. Calculate the number of aggregates in each level from aggregate gradation data.
3. Randomly place the generated particles into a predefined sample with no aggregate overlapping. The generated circles in each level should not overlap with circles in other levels.
4. Approximate the generated circles using unit squares and check aggregate gradation. Complement fine aggregates using unit squares.
5. After all particles are completed, the region within the sample boundary, but not occupied by aggregate, is set as asphalt binder. Air voids are generated by deleting the asphalt binder elements randomly. It should be noted that the actual volume of aggregate is greater than the one used in the model because of the adsorbed portion of the asphalt binder by the aggregate.

**Figure 2:**
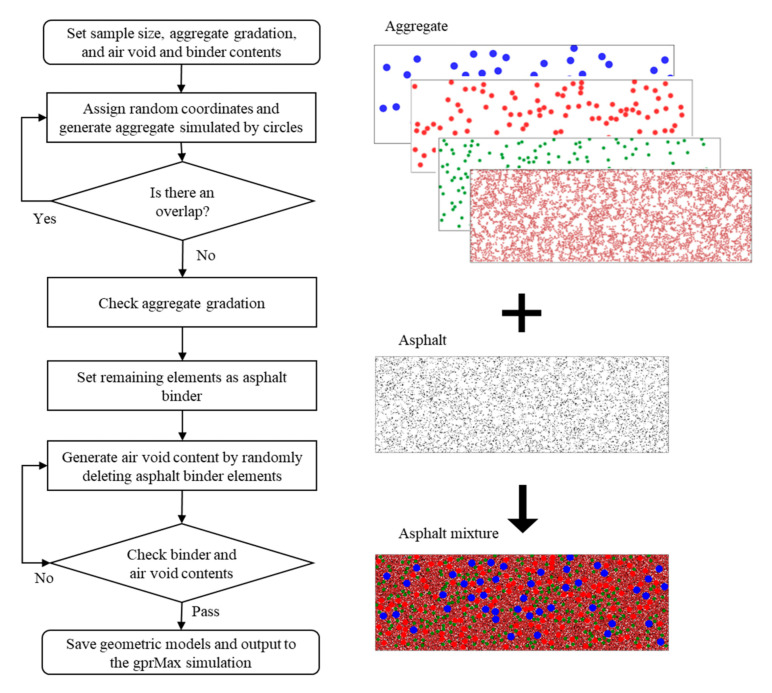
A flowchart of the three-phase asphalt-mixture generation algorithm.

**Figure 3:**
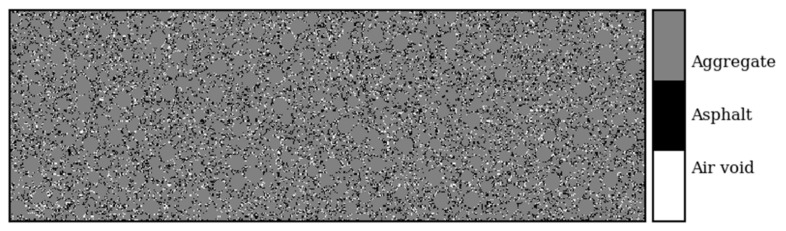
Example of a three-phase asphalt mixture numerical model.

The flowchart of the AC-mixture generating algorithm is shown in Figure 2. The results are saved as a numerical matrix, and they are further used in GprMax simulation. The constructed numerical model of an AC mixture contains three phases: aggregate, asphalt binder, and air voids. An example of the heterogeneous model is shown in Figure 3.

### 3.3. FDTD Simulation Models

**Figure 4:**
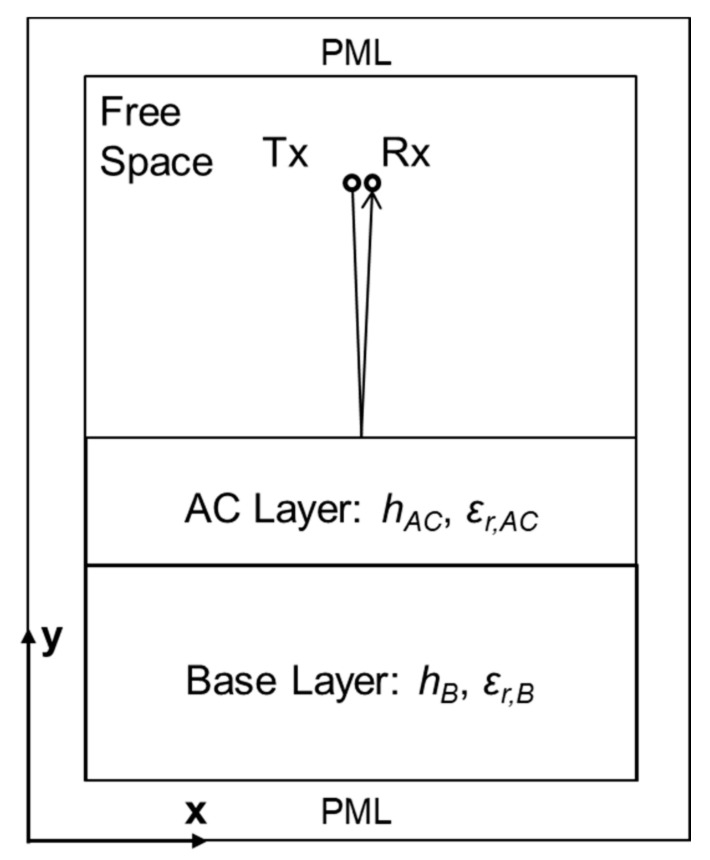
The finite-difference time-domain (FDTD) simulation model in the GprMax.

The diagram of the FDTD model in GprMax is shown in Figure 4. Two layers, including the AC surface layer of height—$h_{AC}$—and base layer of height—$h_{B}$, were built in this model using the AC-mixture generating algorithm. Tx is the transmitter antenna, and Rx is the receiver antenna. The transmitter antenna sends EM waves into the ground, and the receiver antenna can receive reflected signals from the interface between the free space and the AC layer, as well as the interface between the AC layer and the base layer. The perfect matched layer (PML) is used to cancel out any reflections upon its interface. The model with PML can simulate the EM wave propagation in an infinite space.

### 3.4. Generating Heterogeneous Asphalt Mixture Models

Two levels of heterogeneity are discussed in this paper. The first involves a model given a constant air-void value when it is developed. The second includes air voids set differently along the model space. In the proposed three-phase AC mixture generation algorithm, a constant air-void value is given to generate the three-phase numerical models. Heterogeneity is caused by the variation of the three-phase microstructure inside the model.

The air voids, however, change inside the pavement layer in the field. Previous studies showed that air voids change along the depth right after AC compaction [32,33,34]. The air voids could be higher at the top of the AC layer than at its bottom. The air-void distributions along the depth further change because of the compaction from traffic. In addition, AC segregation may exist due to several causes including mix design, unevenness of stockpiling, material mixing, truck loading and unloading, and malfunction of pavers.

**Figure 5:**
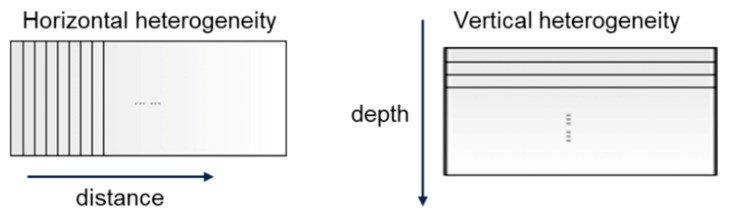
Horizontal and vertical heterogeneity in a simulation model.

To analyze the effect of vertical and longitudinal heterogeneity, a heterogeneous AC, known as the take-and-add generation algorithm, is developed based on the three-phase AC mixture generation algorithm:

1. Generate a three-phase AC mixture model in accordance with the proposed three-phase AC generation algorithm. The air voids of the model can be set at any value between the maximum and minimum air voids of the heterogeneous model.
2. Divide the model into sections. To analyze the effect of longitudinal heterogeneity, the model is divided along the distance/traffic direction. To analyze the effect of the vertical heterogeneity, the model is divided along the pavement depth, as shown in Figure 5.
3. According to the new AC mixture information, binder-coated aggregates were replaced with air void inclusions (i.e., particles) if a higher air void content was required and vice versa.

Another method to generate a heterogeneous AC mixture model is to generate three-phase AC sections with various air void contents and then concatenate them along the longitudinal distance or depth. Compared with the proposed take-and-add method, this method would cause discontinuity at the boundaries of the divided sections [35].

The method for generating a three-phase numerical model with a constant air-void setting is called the three-phase AC generation algorithm, while the one with different air voids along the space is called the heterogeneous AC take-and-add generation algorithm.

## 4. Simulation Results and Discussion

### 4.1. Comparison between One- and Three-Phase AC Models

A comparison was performed between the one-phase model developed by Shangguan and Al-Qadi [8] and the proposed three-phase model. Both models have two layers, including AC surface and base layers. The only difference between the two models is that in the one-phase model, the dielectric constants of the pavement layers are given as single values. In the three-phase model, the dielectric constants and volume properties of air, asphalt binder, and aggregates are given to generate the model.

**Figure 6:**
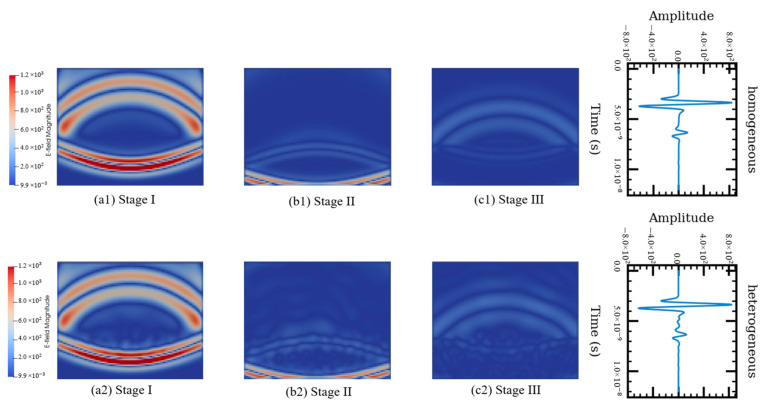
Comparison between the one- and three-phase AC simulation models.

The results are shown Figure 6. The first row shows the electric field magnitude of the one-phase model in different stages and the corresponding received signal. The second row shows the electric field magnitude of the three-phase model in different stages and the corresponding received signal. At stage I, part of the EM waves was reflected from the top of the AC surface layer and the rest transmitted downwards. The reflected waves at stage I correspond to the first wavelet of the received signals shown in Figure 6. The transmitter signal experienced another reflection at the interface between the AC surface layer and the base layer (stage II). This corresponds to the second wavelet on the received signals. At stage III, the reflected signal from the bottom of the AC surface layer is reflected and transmitted again at the top of the AC layer. The EM energy, however, so weak because of multiple-time reflections results in no wavelet being observed on the received signals. In the one-phase model, specular reflection occurs when the EM waves hit the interface of two different materials. In the three-phase model, however, the EM waves were scattered from the surface at different angles rather than at just one angle—as in the case of the one-phase model. Hence, small fluctuations were observed between the first and second wavelets in the received signal rather than a constant value of zero, which is observed from field signals. This suggests that the three-phase AC model is able to capture the wave distortion caused by the microstructure of the AC mixture—compared with the one-phase numerical model.

### 4.2. Field Validation

**Figure 7:**
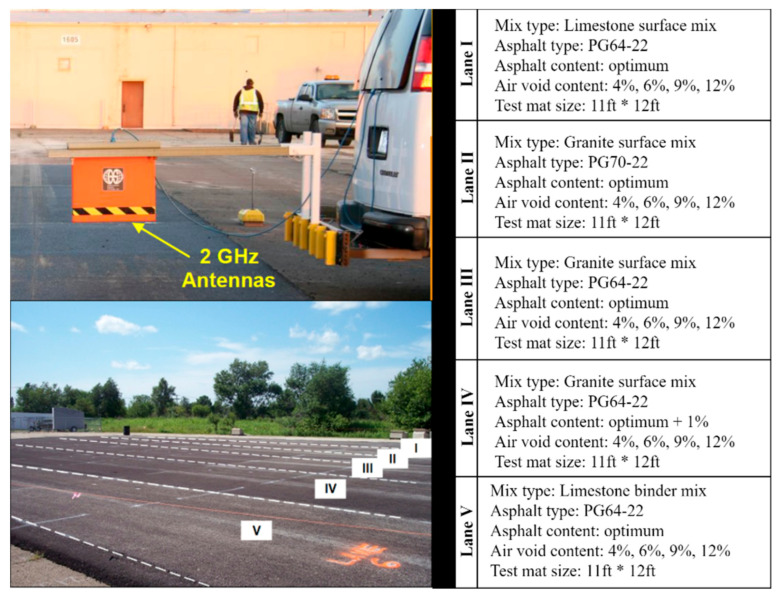
Test sites for validation (after [7]).

The proposed simulation method was validated by field tests data. The test site is a large AC-surfaced parking lot constructed at the Advanced Transportation Research and Engineering Laboratory of the Illinois Center for Transportation at the University of Illinois Urbana-Champaign. The site was used to develop the Al-Qadi-Lahouar-Leng (ALL) model [7]. As shown in Figure 7, five lanes with different AC and asphalt binder types were built. Each lane is comprised of four sections, each has a different air void content. A transition section was placed between the adjacent sections to allow compaction level adjustment. The aggregate gradation of various AC types is shown in Table 1. The same limestone aggregate was in all AC mixes.

A 2 GHz air-coupled, van-mounted GPR system was used to conduct the GPR surveys over each test sections (see Figure 7). The dielectric constants of AC were calculated using Equation (1). The aggregate dielectric constants were back-calculated using 6-in-diameter cores extracted from each lane [7]. Dielectric constants of the AC mixture were calculated using three specific gravity models (see Equations (6)–(8)). Numerical models were constructed with the same air voids, aggregate types, asphalt binder contents, and aggregate gradation as shown in Figure 7 and Table 1.

**Figure 8:**
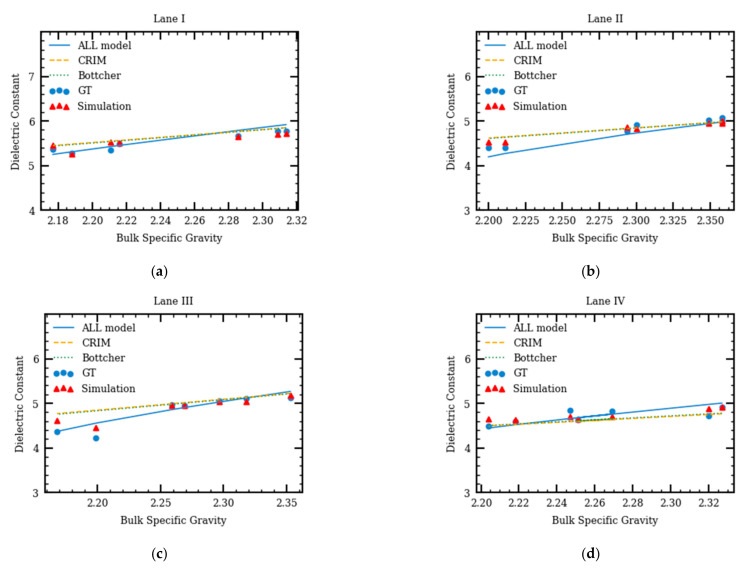

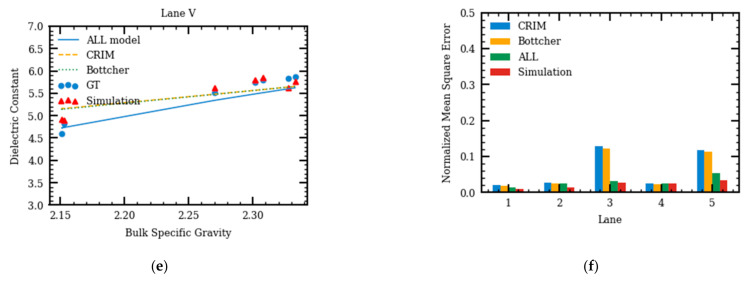
Comparison between ground truth (GT) and results from different models for lanes I–V (**a**–**e**) as well as the normalized mean square error of different models (**f**).

The comparisons among the AC dielectric constant ground truth (GT), dielectric constant calculated using the theoretical specific gravity models, and the ones obtained from the simulation models are shown in Figure 8. The normalized mean square errors are calculated using Equation (9).

(9) $$NMSE=\;\frac{\sum {\left(model\;result-ground\;truth\right)}^{2}}{ground\;truth}$$

Figure 8 suggests that the results from the numerical models are closer to the predictions from the ALL model. The average prediction errors of the complex refractive index model (CRIM), Bottcher, ALL, and simulation models are 6.4%, 6.04%, 3.0%, and 2.2%. Both ALL and simulation models have smaller errors compared to the CRIM and Bottcher models. Compared with the specific gravity models, the proposed numerical model performed well, and results are the closest to the GT. This suggests that the three-phase numerical model can be used to describe the relationship between the dielectric constant of the AC and the dielectric constants and volumetric properties of its components.

### 4.3. Sensitivity Analysis

**Figure 9:**
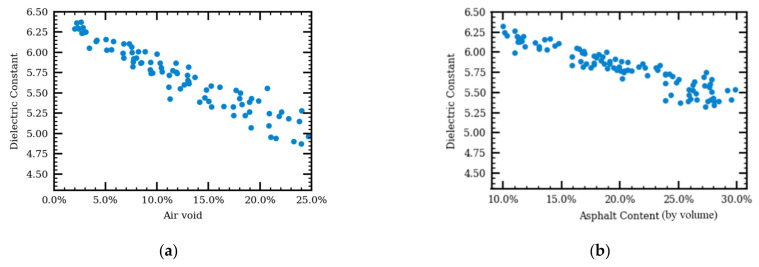
The relationship between dielectric constant and air void (**a**) and the relationship between dielectric constant and asphalt content (**b**).

Sensitivity analysis was performed to analyze the effect of air voids, asphalt contents, and aggregate gradations on the AC’s dielectric constants using the Monte Carlo method. The relationship between the dielectric constants and air voids is shown in Figure 9a. One hundred three-phase simulations with air voids randomly chosen from 2.0% to 25.0% were generated. The asphalt contents were the same for all 100 simulations. The dielectric constants were calculated using Equation (1). It was shown that the dielectric constant decreases with air voids, which is the same conclusion from the theoretical specific gravity models. The relationship between asphalt contents and AC’s dielectric constants is shown in Figure 9b. As would be expected, the higher the asphalt content, the lower the AC dielectric constant for the same air void.

For both relationships shown in Figure 9, variations are observed in the AC predicted dielectric constant given the same air voids or asphalt binder contents. Even for the same air void or asphalt content, the dielectric constant of the AC may differ by ±3% due to AC microstructure variation. This variation is expected to increase with air void and/or asphalt content.

**Figure 10:**
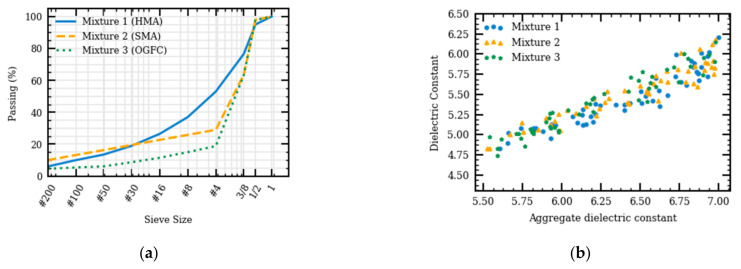
Aggregate gradation for various asphalt concrete (AC) mixtures (**a**) and dielectric constants for various AC mixtures having different aggregate dielectric constants (**b**).

To determine the effect of aggregate gradation on predicted dielectric constant, results from the generated simulations were compared to those obtained from numerical models. Three aggregate gradations, dense-, gap-, and open-graded AC mixtures were simulated in the numerical models. The asphalt content and air void of the three AC mixtures were kept constant. Mixtures 1, 2, and 3 are dense-graded hot mix asphalt (HMA), gap-graded stone matrix asphalt (SMA), and open-graded friction course (OGFC) asphalt concrete mixtures, respectively. The aggregate gradations are shown in Figure 10a. Figure 10b presents the calculated dielectric constants of the three mixes. No obvious differences could be observed among the three different aggregate gradations. This confirms that the AC dielectric constant is mainly affected by its component’s dielectric constants and relative volumes and is independent of the aggregate gradation. Increasing the aggregate dielectric constant would increase the AC dielectric constant, which is in agreement with the sensitivity analysis of the theoretical specific gravity models [7].

### 4.4. Effect of Material Heterogeneity

#### 4.4.1. Vertical Air-Void Changing

**Figure 11:**
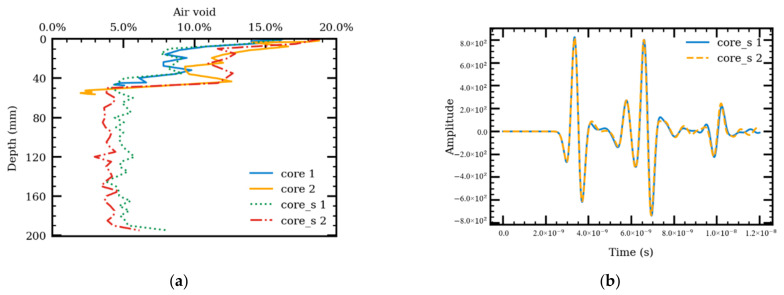
Air void distribution along the depth (**a**) and corresponding received signals from the simulation models (**b**).

To study the effect of vertical heterogeneity, vertical air-void distribution obtained by Masad et al. [32] was used. The air void distributions of two field cores are shown in Figure 11a as cores 1 and 2. The heterogeneous models were generated using the proposed heterogeneous AC’s take-and-add generation algorithm, and the air-void distributions are shown as cores 1 and 2 in Figure 11a. FDTD simulations were performed on the generated models, and the received signals are shown in Figure 11b. Two different methods were used to calculate the AC dielectric constants, including the reflection amplitude and TWTT methods (see Equations (1) and (2)). 

For core 1, the calculated dielectric constant, using the reflection amplitude method, is 4.962, while it is 5.930 using the TWTT method. For core 2, the calculated dielectric constant, using the reflection amplitude method, is 4.890 while it is 5.887 using the TWTT method. In the case of varying vertical air voids, the calculated dielectric constants using the two methods also differ by up to 1.0. In both cases, the results from the reflection amplitude method are lower than the results from the TWTT method. This could be contributed to the effect of shallow-depth air voids on the reflection amplitude method. The TWTT method, however, calculates the average dielectric constants throughout the depth. Both cores have higher air voids/lower density near the surface compared to the bottom. This suggests that the TWTT method is more appropriate; especially when the air void content varies throughout the AC layer depth.

#### 4.4.2. Longitudinal Air-Void Changing

**Figure 12:**
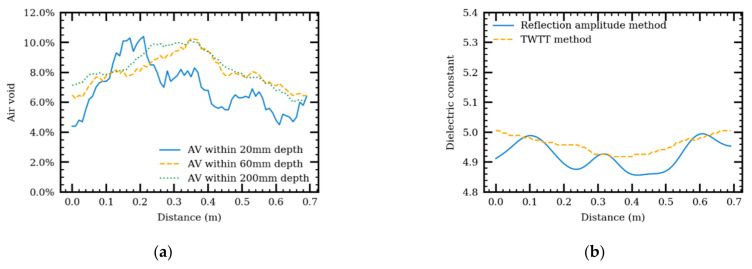
Air void distribution along distance (**a**) and dielectric constant along distance using the reflective amplitude and TWTT methods (**b**).

For longitudinal heterogeneity effect, a model was generated with varying air voids between 6.0% to 10.0% along the GPR survey direction. The generated air-void distribution was validated at different depths—20 mm, 60 mm, and 200 mm—of AC specimen as shown in Figure 12a. The variation of air voids within a 60-mm depth shows a similar trend as the variation of air voids within a 200 mm depth. Air voids within a 20-mm depth, however, have a higher variation. Although constant air voids along the depth were set when the model was constructed, air voids within different depths are not the same because of the variation in the AC microstructure. Comparison between the dielectric constants, calculated by the reflection amplitude method and the TWTT method, is relatively small (0.1), as shown in Figure 12b.

The trend of dielectric constant along the survey direction varies. For example, at 0.3 m distance, the dielectric constant, calculated using the reflection amplitude method, increases, but the dielectric constant, calculated using the TWTT method, decreases. The trend of the dielectric constants, calculated using the reflection amplitude method, is closer to the inverse of the variation of air voids within 20 mm depth. The relationship between air voids and calculated dielectric constants is the same as the relationships shown in Figure 9a. This suggested that dielectric constants using the TWTT method can indicate an overall air void change in the case of longitudinal heterogeneity. On the other hand, dielectric constants using the reflection amplitude method focus on the air-void change in the thin layer close to the pavement surface.

## 5. Conclusions

This paper discusses the development of a heterogenous numerical model to simulate ground-penetrating radar (GPR) wave interaction with pavement structure. The numerical model was used to predict the dielectric constant of asphalt concrete (AC) from dielectric properties of the mix’s three components. Laboratory experiment data were used to verify the numerical simulation results. A take-and-add generation algorithm generated the uneven air void distribution along vertical (depth) or longitudinal (traffic-moving) directions in AC pavement layers. The effects of vertical or longitudinal heterogeneity on the reflected signals were discussed. The findings of this study can be summarized as follows:

1. A three-phase heterogeneous model was introduced to simulate the GPR reflected signal utilizing the dielectric constant and volumetric properties of the AC components: air, asphalt binder, and aggregates. The model predicts the AC density profile, while capturing the AC heterogeneity and its components’ effects.
2. Sensitivity analysis shows that an increase in AC air voids and/or asphalt content would decrease the AC’s dielectric constant, similarly when the aggregate dielectric constant decreases. Aggregate gradation has no effect on the calculated dielectric constant at the GPR frequency and the aggregate sizes used.
3. The calculated method of AC dielectric constant is important. The proposed numerical model is able to simulate uneven air-void distribution in the AC pavement layer. For vertical heterogeneity, the dielectric constant, calculated using reflection amplitude and two-way travel time (TWTT) methods, can differ by 1. The reflection amplitude method is more sensitive to AC density in the shallow layer, while the TWTT method, calculates the average dielectric constants throughout the depth. Hence, the TWTT method is suitable for thick AC pavement layers.

In future research, the variation of AC density and internal moisture content will be applied in the numerical pavement model simultaneously to quantify the moisture in AC.

## Figures and Tables

**Table 1:** Gradation Information of Different Mixture Types (after [7]).

	Passing Ratio (%) for Different Sieving Size										
Mixture type	1	3/4	1/2	3/8	#4	#8	#16	#30	#50	#100	#200
Limestone surface	100	100	100	98.9	58.9	40.0	29.5	18.5	10.6	7.3	6.0
Gravel surface	100	100	100	97.1	58	41.8	30.8	19.0	10.6	7.2	5.8
Limestone binder	100	98.4	77.2	66.6	47.6	37.1	27.1	16.8	9.5	6.3	5.0

## Data Availability

Data is contained within the article.
